# Whole-flock, metaphylactic tilmicosin failed to eliminate contagious ovine digital dermatitis and footrot in sheep: a cluster randomised trial

**DOI:** 10.1136/vr.103625

**Published:** 2016-07-22

**Authors:** J. W. Angell, D. H. Grove-White, H. J. Williams, J. S. Duncan

**Affiliations:** 1 Department of Epidemiology and Population Health, Institute of Infection and Global Health, The University of Liverpool, Leahurst Campus, Neston, Wirral CH64 7TE, UK; 2 Leahurst Farm Animal Practice, The University of Liverpool, Leahurst Campus, Neston, Wirral CH64 7TE, UK

**Keywords:** Sheep, contagious ovine digital dermatitis, Footrot, tilmicosin, whole-flock treatment

## Abstract

The aim of this study was to evaluate the clinical success of whole-flock systemic tilmicosin and enhanced biosecurity in eliminating active contagious ovine digital dermatitis (CODD) from sheep flocks. Thirty flocks in the UK were randomly allocated to receive either treatment as usual (as per the farmer's normal routine) or whole-flock treatment with tilmicosin, together with isolation and extended treatment of clinically affected individuals and isolation and treatment of purchased sheep during the study period. All flocks were visited once at onset of the trial to examine all sheep. One year later, all sheep were re-examined to determine the presence/absence of clinical lesions. The primary outcome was the clinical elimination of CODD from flocks. Secondary outcomes were reduction in prevalence of CODD, clinical elimination of footrot and reduction in prevalence of footrot. The analysis included 11 control flocks and 13 intervention flocks, with initially 3460 and 4686 sheep, respectively. For CODD: at follow-up, in the intervention group, 6/13 (46 per cent) flocks had a prevalence of zero compared with 1/11 (9 per cent) in the control group (P=0.12). For footrot: at follow-up, no flocks had a prevalence of zero. Therefore, the intervention is not recommended for the elimination of CODD or footrot in the UK.

Contagious ovine digital dermatitis (CODD) is now common and causes severe lameness and pathological changes in the foot (Angell and others 2014, 2015a). Clinical lesions have been associated with *Treponema* species phylogenetically identical to those associated with bovine digital dermatitis (BDD) (Sullivan and others 2015), and as such, are currently considered a necessary cause of disease (Duncan and others 2014).

To date, there have been few reports detailing attempts at treating CODD. In Duncan and others (2011, 2012), cure rates of approximately 80 per cent were achieved with systemic amoxicillin and topical chlortetracycline. A case report by Watson (1999) indicated that a single injection of tilmicosin, together with topical oxytetracycline, led to the clinical recovery of a severe ovine foot disease (clinically resembling CODD) in a group of approximately 100 lambs. Furthermore, Judson (2010) reported that systemic oxytetracycline, together with a tylosin footbath before housing, prevented the development of CODD in groups of lambs from an infected flock.

The only in vitro antibiotic sensitivity study of *Treponema* species isolates cultured from CODD lesions demonstrated low minimum inhibitory concentrations (MICs) and minimum bactericidal concentrations (MBCs) for penicillin and macrolide antibiotics (Angell and others 2015b).

Recently, Sawyer (2010) reported the use of a whole-flock treatment with tilmicosin at a dose of 5 mg/kg in 15 flocks affected by footrot and/or CODD. However, the authors did not examine the sheep before treatment, there were no details of disease prevalence/incidence data before or after treatment and there were no control data hampering evaluation of the study. It is recognised that with the high welfare impact of CODD the inherent feasibility issues of individual animal treatment of lame sheep, and the observation that a large proportion of sheep with lesions are not actually lame (Angell and others 2015d), the prospect of elimination via a single mass treatment is attractive to farmers and vets. Indeed, there are widespread, numerous, anecdotal reports of veterinarians across the UK using this regimen.

However, with current concerns over antibiotic use in farm animals (Page and Gautier 2012), this whole-flock approach (advocated by the manufacturer, and based on a poorly designed study; Sawyer 2010) needs to be robustly, scientifically and ethically justified. Therefore, the aim of this current study was to evaluate the clinical success of using whole-flock systemic tilmicosin, together with isolation and repeated treatment of clinical cases, and treatment of purchased sheep, in eliminating active CODD from sheep flocks for one year using a randomised controlled trial methodology.

Tilmicosin is described as a ‘critically important antimicrobial’ (WHO 2011) and is therefore a controversial choice for metaphylaxis. However, it is not excluded from veterinary use, but highlighted as requiring more careful consideration.

Currently, there are no treatments licensed for CODD; therefore, the choice of treatment is empirical. In considering a particular antibiotic to investigate as a therapeutic agent, tilmicosin (Micotil; Elanco Animal Health) was considered for the following reasons:

1. Tilmicosin demonstrated low MIC and MBC data in an *in vitro* study (Angell and others 2015b).
2. Tilmicosin already has a UK licence for the treatment of footrot in sheep, so could be justified in terms of the UK Veterinary Medicines Cascade (DEFRA 2013b).
3. Given the deep pathology associated with the disease (Angell and others 2015a), systemic antibiosis was preferred over topical application.
4. Due to the lack of knowledge in treating CODD, current therapeutic strategies for other treponemal diseases, including BDD and syphilis, were considered. For BDD, topical strategies aimed at control are preferred to systemic antibiosis due to the economic costs associated with milk withholds (Laven and Logue 2006).
  In the treatment of syphilis, the objective is for a clinical cure and treatment can be required for up to 28 days (Kingston and others 2008).
  In the case of CODD, tilmicosin could be expected to persist within infected tissues (Scorneaux and Shryock 1999, Naccari and others 2001), and furthermore, a repeat dose could be given safely after 14 days (Elanco Animal Health, personal communication), thereby potentially achieving a 28-day treatment period.
5. The responsible use of macrolides in veterinary medicine is imperative given that they are considered by the WHO to be critically important for human medicine (WHO 2011). In the UK, tilmicosin injection can only be administered by a veterinary surgeon (Elanco 2013), which may ensure more accurate and responsible use.
6. Tilmicosin was also chosen for this study, despite its status as ‘critically important’, on the basis that successful elimination would potentially result in lower overall use of antimicrobials.
7. Anecdotally there are good clinical responses in treating CODD with tilmicosin (Watson 1999).
8. A pilot study examining the clinical efficacy of tilmicosin showed excellent results. A cohort of 58 affected individuals, with 62 active CODD lesions, were treated at a dose of 10 mg/kg, given subcutaneously twice 14 days apart. The sheep were then re-examined four and six weeks after the first injection. At these visits, all the active lesions had healed, although the horn was thinner and soft in many cases, and there were no new cases or reversions to active CODD.

## Materials and methods

### Study design

The null hypothesis was that a single whole-flock treatment with tilmicosin, together with isolation and repeated treatment of cases and treatment of purchased sheep, would result in the elimination of CODD for one year based on clinical examination and lesion scoring. To test this hypothesis, a cluster randomised controlled trial design was used, with flocks as the cluster units, and control flocks continuing with treatment as usual (TAU).

### Study population

A convenience sample of 30 flocks was recruited (Table 1), the inclusion criteria being having active CODD (lesion grades 1–4; Angell and others 2015a) in the flock as diagnosed by the authors, having a flock size of approximately 300 ewes and being willing to fulfil the requirements of the study. A flock size of 300 ewes was chosen to reflect the average flock size in the UK (excluding smallholders) for 2013 (DEFRA 2013a), and from experience, this number of sheep would be feasible to gather, examine and treat in one study day. Twenty-one flocks were located in Wales, four in Devon, two in Lancashire and one each in Derbyshire, Cheshire and Yorkshire. Each farmer participated voluntarily, provided informed consent and was able to leave the study at any time. No financial payments were made to any of the farmers, but all treatments in both groups were provided free of charge as a gesture of goodwill. In addition, those flocks randomly allocated to receive TAU were given the option to receive the intervention at the end of the trial provided they had remained in the study for the duration.

**TABLE 1:** Attributes of the 30 study flocks in England and Wales as reported at the first visit at the commencement of the study

Flock	County	Land type	Total size (ha)	Total number of breeding ewes (LU*)	Total number of cattle (LU*)	Stocking density: number of livestock units per hectare	Breeds (%)
1	Powys	Lowland	28.3	175 (19.3)	64 (41.6)	2.2	Mules (90.9) Other crossbreeds (9.1)
2	Denbighshire	Lowland	60.7	510 (56.1)	82 (53.3)	1.8	Texel X (31.1) Welsh mule (22.7) Mule (20.0) Suffolk X (13.5) Other crossbreeds (12.7)
3	Conwy	Lowland	78.9	351 (38.6)	120 (78.0)	1.5	Mule (67.2)
							Suffolk X (20.5) Texel X (7.4) Other crossbreeds (4.9)
4	Denbighshire	Upland	80.9	696 (55.7)	90 (58.5)	1.4	Beulah speckled face (96.3) Other crossbreeds (3.7)
5	Denbighshire	Upland	40.5	211 (23.2)	51 (33.2)	1.4	Mule (92.6) Texel (5.5)
							Other crossbreeds (1.9)
6	Devonshire	Lowland	64.8	463 (50.9)	48 (31.2)	1.3	Suffolk (71.4) Mule (21.2) Texel X (3.9) Other crossbreeds (3.5)
7	Powys	Lowland	76.9	381 (27.1)	118 (76.7)	1.3	Texel X (30.2) Mule (27.8) Welsh Mountain X (27.8) Charolais X (8.1) Other crossbreeds (6.1)
8	Yorkshire	Hill	40.5	350 (27.7)	0	0.7	Swaledale (61.7) Mule (17.7) Texel X (16.0) Texel (3.4) Other crossbreeds (1.2)
9	Lancashire/ Cumbria	Upland	131.5	535 (58.9)	52 (33.8)	0.7	Texel X (56.3) Mule (42.2) Other crossbreeds (1.5)
10	Devonshire	Lowland	80.9	441 (48.5)	55 (35.8)	1.0	Mule (38.3) Romney X (26.1) Lleyn X (18.8) Suffolk X (11.1) Other crossbreeds (5.7)
11	Powys	Lowland	22.3	168 (18.5)	140 (91.0)	4.9	Texel X (67.9) Mule (22.6) Suffolk X (7.1) Other crossbreeds (2.4)
12	Denbighshire	Lowland/upland	80.9	414 (42.0)	40 (26.0)	0.8	Aberdale (79.5) Welsh Mountain (16.9) Other crossbreeds (3.6)
13	Conwy	Hill	121.4	248 (14.9)	74 (48.1)	0.5	Welsh Mountain (100.0)
14	Derbyshire	Upland	32.4	228 (23.9)	0	0.7	Texel/Texel X (69.7) Mule (19.7) Swaledale (10.5)
15	Denbighshire	Upland	52.6	379 (30.4)	0	0.6	Welsh Mountain X (99.5) Charolais X (0.5)
16	Powys	Hill/upland	64.8	250 (27.5)	0	0.4	Texel X (45.6) Suffolk X (24.4) Lleyn X (22.0) Mule (6.4) Charolais X (1.6) Border Leicester (1.0)
17	Denbighshire	Lowland	48.6	359 (38.1)	10 (6.5)	0.9	Suffolk X (24.2) Texel X (21.7) Mule (19.5) Welsh Mountain X (12.8) Charolais X (11.7) Other crossbreeds (10.1)
18	Cheshire	Lowland	40.5	315 (34.7)	0	0.9	Texel X (68.2) Cheviot X (28.7) Other crossbreeds (3.1)
19	Conwy	Lowland	32.4	343 (37.7)	0	1.2	Texel X (62.1) Suffolk X (30.6) Mule (7.3)
20	Conwy	Upland	44.9	259 (28.5)	60 (39.0)	1.5	Texel X (100)
21	Conwy	Upland	80.9	384 (38.9)	75 (48.8)	1.1	Mule (65.9) Welsh Mountain X (14.8) Welsh Mountain (8.3) Texel X (7.3) Other breeds (3.7)
22	Denbighshire	Lowland	70.8	410 (43.9)	55 (35.8)	1.1	Cheviot X (37.8) Texel X (19.7) Mule (15.2) Romney X (13.3) Welsh Mountain X (9.6) Other crossbreeds (4.4)
23	Devonshire	Lowland	48.6	116 (12.8)	40 (26.0)	0.8	Suffolk X (73.0) Mule (20.0) Lleyn X (3.5) Other crossbreeds (3.5)
24	Denbighshire	Lowland	121.4	338 (37.2)	100 (65.0)	0.8	Lleyn X (97.0) Other breeds (3.0)
25	Devonshire	Lowland	64.8	317 (34.9)	10 (6.5)	0.6	Mule (100)
26	Conwy	Upland	123.4	287 (31.6)	70 (45.5)	0.6	Lleyn X (43.2) Suffolk X (21.6) Polled Dorset (16.0) Mule (14.3) Texel X (3.5) Other breeds (1.4)
27	Conwy	Upland	121.4	491 (37.4)	53 (34.5)	0.6	Welsh Mountain (53.4) Texel X (31.0) Welsh Hill speckled face (14.3) Other breeds (1.3)
28	Powys	Lowland	32.4	184 (20.2)	150 (97.5)	3.6	Texel X (100)
29	Lancashire	Lowland		600	This farm dropped out before the first visit due to personal reasons		
30	Conwy	Upland		400	This farm dropped out before the first visit as the ewes were in poor condition		

*Livestock units (LU): Lowland ewes 0.11, Upland ewes 0.08, Hill ewes 0.06, Cattle 0.65 (DEFRA, 2010)

### Interventions and follow-up

Flocks were randomised to the control or intervention arms after recruitment using simple randomisation with random numbers. All the flocks were visited once at the beginning of the study to establish baseline prevalence data and administer the intervention, and then on one further occasion a year later to record the end prevalence data. In addition, they were visited a further three times evenly spaced in the interim period (at approximately three-month intervals) in order to monitor the health of the ewes and to retain contact with the farmers.

For both the initial visit and final visit, each flock was visited on a single day at a point approximately in mid gestation. This was considered the safest time to examine pregnant sheep, and also when fewest sheep would be present on the farm—mainly ewes and rams—ensuring the minimum amount of antibiotic used. Furthermore, it was considered that CODD prevalence might be at a lower level at this time than at other times of year (Angell and others 2015d), which might make elimination more likely. At the initial visit, every sheep on the farm was individually examined and data recorded.

For the control group, any foot lesion observed (of any type, e.g. footrot, CODD, foot abscess) was treated as per the farmers’ normal routine as agreed with their own veterinary surgeon. For the intervention group, the intervention comprised both treatment of all animals together with extended treatment and isolation of all clinical cases. All sheep received a single dose of tilmicosin (Micotil, Elanco Animal Health) at an estimated dose of 10 mg/kg bodyweight administered subcutaneously over the left shoulder via an automatic injector through a ¾ in.×18 g needle. In addition, any sheep observed to have clinically active CODD (lesion grade 1–4; Angell and others 2015a) was marked and isolated in a separate group on the farm. A second dose of tilmicosin was then administered to these individual sheep 14 days later in exactly the same manner as the first dose, except that it was given over the right shoulder. The farmer was then instructed to continue the isolation of these individuals for a further 14 days, that is, affected sheep were isolated for 28 days post initial treatment. Furthermore, throughout the year farmers were instructed to isolate any sheep moved on to the farm, for example, replacement ewes or rams. Those sheep were then inspected by the author and treated with a single dose of tilmicosin at this point, and the farmers were then instructed to keep those new animals in isolation for 14 days.

### Outcomes

The primary outcome was the clinical elimination of active CODD at the final visit. A further secondary outcome was the reduction in prevalence of active CODD. A flock was deemed to have active CODD if any single foot was observed with an active lesion in that flock. Healed lesions (grade 5) could be present. With regard to active CODD, any lesion deemed to be ambiguous (n=2) was biopsied and the tissue tested by nested PCR assays specific for any of the three CODD-associated treponeme phylogroups: *Treponema medium/Treponema vincentii*-like, *Treponema phagedenis*-like and *Treponema pedis* (Sullivan and others 2015) to confirm the diagnosis.

Due to the data available, the same analyses could also be done for footrot. Footrot was diagnosed as inflammation and erosion/ulceration of the interdigital skin together with underrunning of the hoof horn axially and onto the sole (Egerton and Roberts 1971). In this case, the primary outcome was the clinical elimination of footrot at the final visit and a secondary outcome was the reduction in prevalence of footrot.

Foot lesions were classified on the basis of their clinical appearance as CODD together with grade as per Angell and others (2015a), footrot as per the description by Egerton and Roberts (1971) and also used by Foddai and others (2012) and foot abscess as described by West (1989).

In some cases, feet could be considered to have features of both footrot and CODD, or scald and CODD. In these cases, the combination was recorded. Careful observation allowed key diagnostic features to be observed.

For footrot, there was always an interdigital dermatitis (ID) lesion present, and the lesion distribution was axial and frequently involved the solar horn.

For clinically active CODD, lesions appeared to commence at the coronary band (compared with the interdigital space as in footrot) and could be distinguished from footrot lesions as they frequently did not have an axial lesion and appeared to ‘shell out’ the hoof horn capsule as opposed to ‘digest’ it from an external origin.

Foot abscesses were observed to have burst out at the coronary band and were distinguished from an early CODD lesion by the presence of frank pus and a cavity subjacent to the visible lesion revealed through careful examination. Confusion with early CODD lesions was eliminated by distinguishing whether there was erosion/ulceration of the coronary band compared with the voiding of pus from a cavity, and confusion with later CODD lesions was eliminated by determining the extent of the underrunning of the hoof horn that typically is narrow and confined to the tracking of the pus in a foot abscess rather than widespread tending to circumferential underrunning as in a clinically active CODD lesion. All sheep were locomotion scored before examination using Angell and others (2015c).

### Sample size calculation

Sample size calculations were carried out for a cluster-randomised design (Hayes and Bennett 1999). At the end of the study, the expected prevalence of CODD in the control group was assumed to be 4 per cent (mean obtained from Angell and others 2014) and in the intervention group 0.5 per cent; the cluster size was 300. The intraclass correlation coefficient was unknown but a coefficient of variation was estimated to be 0.25, power was set at 80 per cent and α 0.05. In order to account for the intracluster correlation, and for an expected 10 per cent drop out of flocks, balanced with the ethical considerations of including enough flocks but no more, 15 farms were recruited to each arm.

### Statistical methods

#### Outcome: elimination of CODD from flocks

The proportion of flocks in the intervention arm that had a CODD prevalence of zero at the final visit was compared with the proportion in the control arm using Fisher's exact test. The effect of the presence of cattle on farms as a potential confounder was investigated using Fisher's exact test and logistic regression.

#### Outcome: change in prevalence of active CODD and footrot

For the outcomes: active CODD and footrot, the prevalence (calculated as the number of sheep with a specific lesion as a percentage of the total flock size) at the initial visit (p1) and at the final visit (p2) were calculated for each farm directly. The mean p1 and p2 for each foot lesion was calculated, adjusting for clustering at flock level. The change in prevalence (p_diff_) was calculated as p1–p2 and displayed graphically. The mean p_diff_ adjusted for clustering at flock level was calculated for the intervention flocks and for the controls flocks together with a 95% CI using robust standard errors; a comparison of these 95% CIs was used to assess the presence of a meaningful difference. Ethical approval was provided by the University of Liverpool ethics committee: VREC13.

## Results

### Recruitment and participant flow

**FIG 1:**
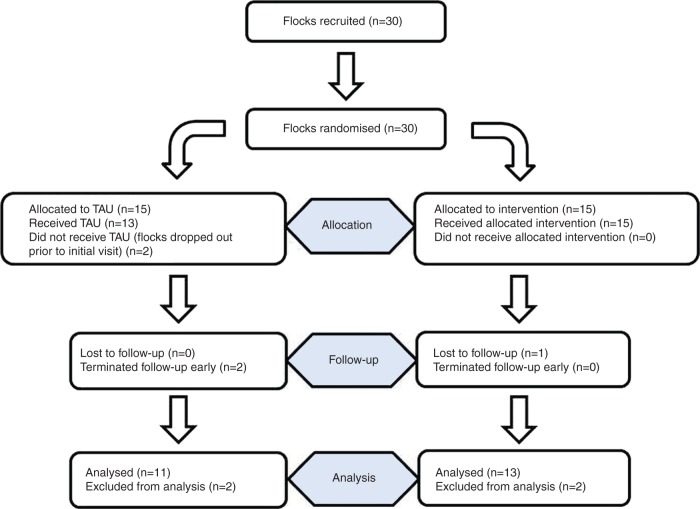
Participant flow diagram detailing the farms included at each stage of the trial. TAU, treatment as usual

Thirty flocks were enrolled in the study during October and November 2013 (Table 1 and Fig 1). Two flocks (29 and 30) dropped out from the control group shortly before their first visit and before being informed of their treatment allocation. Farmer 29 chose not to take part for practical reasons, and farmer 30 was concerned regarding the stress to his animals due to their poor condition. For all flocks, the initial visit occurred between December 3, 2013, and January 29, 2014. All flocks remaining in the study for the duration were visited for the final time (visit 5) between November 21, 2014, and February 13, 2015.

Two control flocks (16 and 19) were excluded from the analysis as the follow-up period had to be terminated two months early (due to personal reasons). Two intervention flocks (4 and 11) were excluded from the analysis due to inadequate follow-up data; farmer 4 failed to present all the sheep for examination at the final visit, and farmer 11 left the study after eight months due to a change of business.

As such, in the final analysis there were 11 control flocks and 13 intervention flocks. At the initial visit, the number of sheep examined in total on the 11 control flocks was 3460 and on the 13 intervention flocks 4686. At the final visit, there were 4354 and 5098 sheep examined, respectively.

### Interventions

#### Control group

At the initial visit, for the infectious foot diseases CODD, footrot and ID, each flock used slightly different combinations of treatments (Table 2). Nine flocks used long-acting oxytetracycline injection (Alamycin LA; Norbrook), three flocks used long-acting amoxicillin injection (Betamox LA; Norbrook), eight used oxytetracycline spray (Alamycin; Norbrook) topically, one used lincomycin and spectinomycin (Lincospectin; Zoetis Animal Health) in a handheld sprayer and one used a tylosin (Tylan; Elanco Animal Health) footbath. For all flocks, feet were only trimmed if there was obvious impingement of soft tissues by loose horn or in some cases of white line disease.

**TABLE 2:** The different combinations of treatments used on each of the different control farms

Flock ID	Oxytetracycline injection	Oxytetracycline spray	Amoxicillin injection	Tylosin footbath	Lincomycin/spectinomycin in handheld sprayer
17	✓	✓			
18	✓			✓	
20		✓	✓		
21	✓	✓			
22	✓	✓			
23	✓	✓			
24	✓	✓			
25			✓		
26	✓	✓			
27	✓	✓	✓		
28	✓				✓

#### Intervention group

At the initial visit, all flocks received the intervention as described.

Baseline data showing the characteristics of each group at flock level (Table 3) reveal significant differences between the intervention and control flocks for footrot only—there being a greater prevalence of footrot for the intervention flocks compared with the controls (P=0.002).

**TABLE 3:** Baseline characteristics of the control (n=11) and intervention (n=13) flocks as recorded at visit 1

	Control (n=11 flocks)			Intervention (n=13 flocks)			
Variable		Flock-adjusted prevalence (%)	95% CI*		Flock-adjusted prevalence (%)	95% CI*	P value†
Foot lesions							
CODD active		2.86	1.99 to 4.09		2.11	1.42 to 3.13	0.3
CODD healed		5.78	4.31 to 7.72		4.61	2.91 to 7.24	0.4
Footrot		12.14	8.60 to 16.87		30.24	19.17 to 44.20	0.002
Scald		12.34	6.55 to 22.05		10.46	4.83 to 21.19	0.7
Granuloma		0.52	0.37 to 0.74		0.19	4.9e^−4^ to 0.76	0.1
White-line disease		22.95	15.45 to 32.67		32.50	21.55 to 45.77	0.2
Foot abscess		0.20	0.10 to 0.41		0.19	0.10 to 0.36	0.9
Interdigital hyperplasia		0.81	0.31 to 2.09		0.38	0.17 to 0.85	0.2
Overgrown		2.20	1.18 to 4.04		1.11	0.53 to 2.30	0.1
Lame		9.19	4.91 to 16.57		13.32	8.20 to 20.91	0.3
Flock variables	n flocks	% flocks	95% CI	n flocks	% flocks	95% CI	P value‡
Farm size (ha)							
Small (28.3–52.6)	5	45.45	16.83 to 77.42	5	38.46	14.64 to 69.48	0.7
Medium (60.7–78.9)	2	18.18	3.45 to 58.00	4	30.77	10.21 to 63.45	0.5
Large (80.9–131.5)	4	36.36	11.67 to 71.20	4	30.77	10.21 to 63.45	0.8
Land type							
Hill	0	0	–	2	15.38	0.00 to 35.00	0.2
Upland	4	36.36	7.93 to 64.79	5	38.46	12.01 to 64.91	0.9
Lowland	7	63.64	35.21 to 92.07	6	46.15	19.05 to 73.25	0.4
Cattle	10	90.91	73.92 to 100	10	76.92	54.02 to 99.82	0.4
Breed							
Upland	8	72.73	46.41 to 99.05	10	76.92	54.02 to 99.82	0.8
Lowland	11	100	–	12	92.31	77.83 to 100	0.4
		Flock-adjusted proportion (%)	95% CI*		Flock-adjusted proportion (%)	95% CI*	P value†
Age							
Lamb		7.39	2.87 to 17.76		17.97	8.45 to 34.21	0.2
1 year		14.46	9.54 to 21.33		15.32	11.45 to 20.20	0.2
Adult		78.14	67.76 to 85.88		66.72	50.71 to 79.61	0.2

For the foot lesions and for age, the flock-level prevalence/proportion (%) has been adjusted to account for the clustering at flock level for each group—control and intervention. For the other flock variables, the percentage of the control flocks and intervention flocks for each variable is calculated directly

*These 95% CIs are calculated using robust standard errors

†P value from a test of independence comparing the farm-adjusted mean farm prevalence for the control farms with that for the intervention farms for each variable using the Pearson χ^2^ statistic with the Rao and Scott (1981, 1984) second-order correction

‡P value from a two-sample test of proportions of flocks comparing the proportion of control flocks with the proportion of intervention flocks for each variable

CODD, contagious ovine digital dermatitis

### Contagious ovine digital dermatitis

#### Outcome: elimination of CODD from flocks

The null hypothesis was that a single whole-flock treatment with tilmicosin, together with isolation and repeated treatment of cases and treatment of purchased sheep, would result in the elimination of CODD for one year based on clinical examination and lesion scoring. Of the control flocks (n=11), one flock (flock 25) (9 per cent) had a prevalence of active CODD of zero at the final visit. Of the intervention flocks (n=13), six flocks (46 per cent) had a prevalence of zero at the final visit. Fisher’s exact test of these two proportions showed no evidence of a difference (P=0.12). None of the farmers of the flocks that had received the intervention and had a final prevalence of zero at the final visit had any observed clinical cases throughout the year following the initial visit. The farmer of flock 25 (control group) had observed some clinical cases during the year but had eliminated these using individual treatment with long-acting amoxicillin injection and culling of some cases.

There was no association between the presence of cattle on the farm and the elimination of CODD at flock level in either the control or intervention groups.

#### Outcome: change in prevalence of CODD

**FIG 2:**
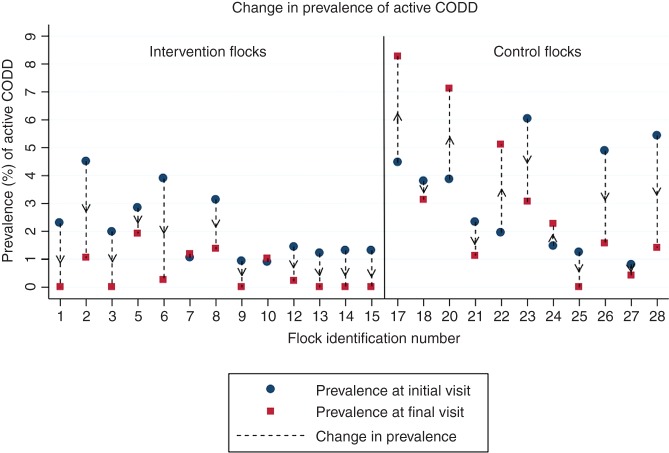
Change in prevalence (p_diff_) of active contagious ovine digital dermatitis (CODD) for each flock between the initial visit and the final visit one year later. Intervention flock: 1–15; control flock: 17–28

The null hypothesis was that there would be no difference between the p_diff_ of active CODD in the control flocks compared with the intervention flocks. Figure 2 (see online supplementary Table S1) shows that at the final visit the prevalence was reduced on 11/13 (85 per cent) of the intervention flocks with no meaningful change in prevalence occurring in two flocks (7 and 10). However, for the control flocks, seven (64 per cent) showed a reduction in prevalence while four (36 per cent) showed an increase in prevalence. The flock-adjusted mean p2 for the control flocks was 2.89 per cent (95% CI 1.64 per cent to 5.03 per cent) and for the intervention flocks was 0.55 per cent (95% CI 0.29 per cent to 1.02 per cent). The flock-adjusted mean p_diff_ for the control flocks was 0.26 per cent (95% CI −1.58 to 2.09) and for the intervention flocks was 1.52 per cent (95% CI 0.84 to 2.21). A comparison of the 95% CIs for these two proportions shows no evidence of a significant difference.

10.1136/vr.103625.supp1 Supplementary table The initial prevalence, final prevalence and change in prevalence for each flock with regard to active CODD and footrot.

### Footrot

#### Outcome: elimination of footrot from flocks

No flocks in either group had a prevalence of footrot of zero at the final visit.

#### Outcome: change in prevalence of footrot

**FIG 3:**
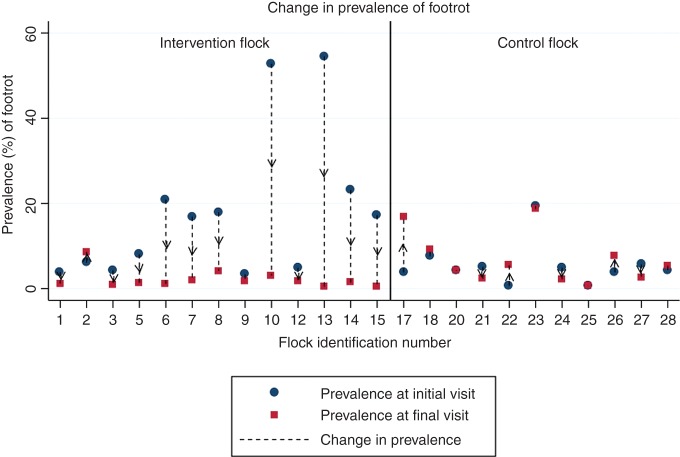
Change in prevalence (p_diff_) of footrot for each flock between the initial visit and the final visit one year later. Intervention flock: 1–15; control flock: 17–28

The null hypothesis was that there would be no difference between the p_diff_ of footrot on the control farms compared with the intervention flocks. Figure 3 (see online supplementary Table S1) shows that all the intervention flocks except farm 2 had a reduced prevalence at the final visit compared with the initial visit. However, of the control flocks, six (55 per cent) showed a reduction in prevalence while five (45 per cent) showed an increase in prevalence. The flock-adjusted mean p2 for the control flocks was 15.37 per cent (95% CI 10.08 per cent to 22.72 per cent) and for the intervention flocks was 6.43 per cent (95% CI 3.68 per cent to 11.00 per cent). The flock-adjusted mean p_diff_ for the control flocks was −2.91 per cent (95% CI −9.93 to 4.12) and for the intervention flocks was 26.05 per cent (95% CI 11.27 to 40.84). A comparison of the 95% CIs for these two proportions shows evidence of a significant difference.

### Harms

During the study, out of all the sheep treated, two died shortly after being injected with tilmicosin, although it is not known whether they died as a result of tilmicosin toxicity. It is feasible that inadvertent intravenous delivery of the drug occurred as a result of the use of an automatic injector, which is a known risk. No other sheep suffered any injury or death associated with the study, and no other effects of either the mass treatments or two examinations were reported.

## Discussion

### Study design and limitations

Table 3 demonstrates that there was a significant difference between the intervention and control farms for the initial prevalence of footrot, despite randomisation. Footrot prevalence was higher in the intervention flocks (30.24 per cent v 12.1 per cent, P=0.002). This was considered to be due to chance owing to the small number of flocks included in the study and is a known risk with cluster randomised trials with small numbers of clusters (Kirkwood and Sterne 2003).

The study was also weakened by a lack of blinding, which was not possible due to practical constraints. As such, this may lead to bias in that farmers in the intervention arm may manage their flocks differently due to being in the intervention arm, and the observer might have been less likely to diagnose a lesion/record lameness in an intervention flock at follow-up. To try and reduce this bias, rigorous observer training was employed with ambiguous CODD lesions biopsied for nested PCR analysis for CODD-associated treponemes.

Further bias is likely to be present due to the convenience sample necessary for this study, in that relatively interested farmers were more likely to be included. However, the study flocks were all commercial flocks with a variety of different breeds, location and land type, and as such, this should strengthen the generalisability of the results.

Interspecies disease transmission, for example, between cattle infected with BDD and sheep, has to the authors’ knowledge never been clearly observed or documented experimentally, although it was hypothesised as a risk factor in Angell and others (2014). As such, due to practical limitations, the BDD status of cattle on the farms was not identified, although this might have strengthened the study in terms of their consideration as a possible reservoir of pathogenic *Treponema* species. In addition, in this study, no association was found between the presence of cattle on the farm and the elimination of CODD at flock level.

### Contagious ovine digital dermatitis

CODD was eliminated from only 6 of the 13 intervention flocks, and one of the control flocks. Furthermore, a comparison of the proportion of flocks that eliminated CODD between the two trial arms was not significant. Where clinical elimination occurred, it was not possible to say whether disease was *eradicated*; however, elimination seems possible since no clinical cases were observed for one year and the observed prevalence at the final visit was zero. Therefore, while the elimination of clinically active CODD at flock level seems possible, there was a high failure rate, and in this study, was no more likely for the intervention farms and was also possible without whole-flock treatment (e.g. farm 25, Fig 2).

Given the need for veterinary practitioners to use antimicrobials responsibly—in particular, macrolides—categorised as critically important for human medicine (WHO 2011), together with the financial costs of such an intervention, then on the basis of this study metaphylactic administration cannot be justified for the elimination of CODD from flocks. Furthermore, the change in prevalence was not significantly different between the intervention and control flocks, suggesting that any initial improvement seen was short-lived and thus this intervention would not be suitable as a control measure.

As demonstrated, even with regular contact and monitoring 7 of the 13 intervention flocks remained affected with active CODD after one year. It is not known why these failures occurred but possible reasons include failure of treatment, persistence of infection in the environment, presence of carrier animals or lapses in biosecurity allowing reintroduction. On discussions with the farmers involved, for the intervention of flocks that failed to eliminate CODD, possible biosecurity issues (e.g. poor fencing) were considered as potential reasons for failure. It is well recognised that farmers may not always follow advice even if given with the aim of helping them (Kaler and Green 2013, Garforth 2015). All the farmers in this study were repeatedly reminded about the need for rigorous biosecurity measures, for example, maintaining adequate fencing, isolating and inspecting purchased animals. However, even with good intentions failings may occur for a variety of reasons.

While the intervention as described failed to eliminate CODD, a high clinical cure rate was observed in the pilot study (100 per cent) as reported in the introduction section. This suggests that tilmicosin may be considered suitable for the individual treatment of sheep with lesions. However, a long-acting amoxicillin preparation also achieved high cure rates (approximately 71 per cent) (Duncan and others 2011, 2012) and would provide a suitable alternative. Given that a significant proportion of sheep with lesions do not show lameness (Phythian and others 2013, Angell and others 2015d), an approach inspecting every foot on the farm, together with the treatment and isolation of clinically affected individuals using one of these two antibiotics, may be more appropriate.

### Footrot

The point prevalence of footrot on some farms was very high, for example, 85 per cent in flock 13 at the initial visit. This was surprising given the average reported prevalences from other studies, for example, 8.3 per cent in Grogono-Thomas and Johnston (1997), 9.4 per cent in Wassink and others (2003) and 3.1 per cent in Winter and others (2015). In the present study, footrot was not eliminated from any flocks, and while there was a significantly reduced prevalence in the intervention flocks compared with the control flocks, the intervention cannot be recommended for this effect. In the report by Sawyer (2010), it was reported that many farms ‘reported no sign of footrot’. However, it is difficult to believe that there were no cases at all given that the author was relying on farmer reports and did not examine sheep at follow-up. Currently, there are well-researched and established effective treatment and control methods for footrot, for example, Clements and Stoye (2014), Green and others (2007), and Wassink and others (2010), and therefore, the use of whole-flock tilmicosin for treating or controlling footrot cannot be justified or recommended for similar reasons as those for CODD.

## Conclusions

After one year, there was no significant difference in clinical elimination of CODD or footrot between the control and treatment groups, nor a significant difference in reduction of CODD prevalence between the two groups. Therefore, the whole-flock macrolide treatment intervention as described cannot be recommended for the elimination of CODD or footrot in UK flocks.
