# Supplementary material for: Whole-flock, metaphylactic tilmicosin failed to eliminate contagious ovine digital dermatitis and footrot in sheep: a cluster randomised trial
Source: Vet Rec. 2016 Jul 22;179(12):308. doi: 10.1136/vr.103625 (PMC5099180; doi:10.1136/vr.103625)
Supplement: Supplementary table — The initial prevalence, final prevalence and change in prevalence for each flock with regard to active CODD and footrot. [file vetrec-2015-103625supp_table.pdf]

	Flock ID number	CODD			Footrot		
		Initial prevalence (%)	Final (%)	Change in (%)	Initial (%)	Final (%)	Change in (%)
Intervention flocks	1	2.29	0	2.29	9.14	3.32	5.83
	2	4.51	1.04	3.47	14.90	18.58	-3.67
	3	1.99	0	1.99	13.96	2.01	11.96
	5	2.84	1.92	0.92	23.70	4.33	19.37
	6	3.89	0.26	3.63	27.86	2.55	25.31
	7	1.05	1.20	-0.15	29.13	5.50	23.63
	8	3.14	1.36	1.78	37.43	13.32	24.11
	9	0.93	0	0.93	9.72	4.40	5.32
	10	0.91	1.03	-0.12	70.29	8.59	61.70
	12	1.45	0.22	1.23	14.25	5.03	9.22
	13	1.21	0	1.21	85.48	1.38	84.10
	14	1.32	0	1.32	46.93	4.37	42.56
	15	1.32	0	1.32	30.34	1.12	29.22
	Flock adjusted mean	2.11 (1.42-	0.55%	1.52% (0.84-	30.24	6.43 (3.68-	26.05 (11.27-
Control flocks	17	4.46	8.28	-3.82	11.14	40.40	-29.26
	18	3.81	3.14	0.67	19.37	19.32	0.04
	20	3.86	7.10	-3.24	14.67	13.07	1.60
	21	2.34	1.13	1.21	11.46	7.24	4.22
	22	1.95	5.11	-3.16	2.44	15.67	-13.23
	23	6.03	3.05	2.98	36.21	38.55	-2.34
	24	1.48	2.26	-0.78	11.54	7.14	4.40
	25	1.26	0	1.26	1.89	2.32	-0.43
	26	4.88	1.57	3.31	11.50	17.45	-5.95
	27	0.81	0.43	0.39	16.70	8.09	8.62
	28	5.43	1.42	4.02	13.59	13.21	0.38
	Flock adjusted mean	2.86 (1.99-	2.89%	0.26% (-1.58-	12.14 (8.60-	15.37	-2.91 (-9.93-

Supplementary Table 1: The initial prevalence, final prevalence and change in prevalence for each flock with regard to active CODD and footrot.
